# Climate variability and *Aede*s vector indices in the southern Philippines: An empirical analysis

**DOI:** 10.1371/journal.pntd.0010478

**Published:** 2022-06-14

**Authors:** Amanda K. Murphy, Ferdinand V. Salazar, Ryan Bonsato, Gemma Uy, Antonietta P. Ebol, Royfrextopher P. Boholst, Callan Davis, Francesca D. Frentiu, Hilary Bambrick, Gregor J. Devine, Wenbiao Hu

**Affiliations:** 1 School of Public Health and Social Work, Faculty of Health, Queensland University of Technology, Brisbane Australia; 2 Mosquito Control Laboratory, Department of Population Health, QIMR Berghofer Medical Research Institute, Brisbane, Australia; 3 Department of Medical Entomology, Research Institute for Tropical Medicine (RITM), Manila, The Philippines; 4 Department of Health, Center for Health Development 10, Northern Mindanao, Cagaya de Oro, The Philippines; 5 Department of Health, Center for Health Development 11, Davao City, Davao del Sur, The Philippines; 6 Department of Health, Center for Health Development Soccskargen Region, Cotabato City, The Philippines; 7 Centre for Immunology and Infection Control, School of Biomedical Sciences, Queensland University of Technology, Brisbane, Australia; Centers for Disease Control and Prevention, UNITED STATES

## Abstract

**Background:**

Vector surveillance is an essential public health tool to aid in the prediction and prevention of mosquito borne diseases. This study compared spatial and temporal trends of vector surveillance indices for *Aedes* vectors in the southern Philippines, and assessed potential links between vector indices and climate factors.

**Methods:**

We analysed routinely collected larval and pupal surveillance data from residential areas of 14 cities and 51 municipalities during 2013–2018 (House, Container, Breteau and Pupal Indices), and used linear regression to explore potential relationships between vector indices and climate variables (minimum temperature, maximum temperature and precipitation).

**Results:**

We found substantial spatial and temporal variation in monthly *Aedes* vector indices between cities during the study period, and no seasonal trend apparent. The House (HI), Container (CI) and Breteau (BI) Indices remained at comparable levels across most surveys (mean HI = 15, mean CI = 16, mean BI = 24), while the Pupal Productivity Index (PPI) was relatively lower in most months (usually below 5) except for two main peak periods (mean = 49 overall). A small proportion of locations recorded high values across all entomological indices in multiple surveys. Each of the vector indices were significantly correlated with one or more climate variables when matched to data from the same month or the previous 1 or 2 months, although the effect sizes were small. Significant associations were identified between minimum temperature and HI, CI and BI in the same month (R^2^ = 0.038, p = 0.007; R^2^ = 0.029, p = 0.018; and R^2^ = 0.034, p = 0.011, respectively), maximum temperature and PPI with a 2-month lag (R^2^ = 0.031, p = 0.032), and precipitation and HI in the same month (R^2^ = 0.023, p = 0.04).

**Conclusions:**

Our findings indicated that larval and pupal surveillance indices were highly variable, were regularly above the threshold for triggering vector control responses, and that vector indices based on household surveys were weakly yet significantly correlated with city-level climate variables. We suggest that more detailed spatial and temporal analyses of entomological, climate, socio-environmental and *Aedes*-borne disease incidence data are necessary to ascertain the most effective use of entomological indices in guiding vector control responses, and reduction of human disease risk.

## Author summary

In areas which regularly experience outbreaks of mosquito-borne diseases, such as dengue, mosquito abundance and climate are two influencing factors which are often explored for their ability to aid prediction of and response to outbreaks. In 65 areas of the southern Philippines which regularly report dengue outbreaks, we compared trends in four different indicators of *Aedes* mosquito abundance during a 6-year period. We also compared data for *Aedes* indicators with climate data from the same areas. We found that patterns in *Aedes* mosquito abundance were highly variable over time and did not appear to have a distinct seasonal trend. The four different indicators of mosquito abundance each had weak yet significant relationships with different climate variables. We propose that further studies should explore additional types of data such as epidemiological and socio-environmental data for their value in assisting public health responses to *Aedes*-borne disease outbreaks.

## Introduction

In countries endemic for *Aedes*-borne diseases, public health surveillance commonly includes entomological surveys to assess the presence and abundance of mosquito vector species. Survey methods often focus on identifying the immature mosquito stages, the larvae or pupae, present in breeding containers within and around homes [1]. Information gathered through surveys can be used to characterise and monitor mosquito species distributions, and as an indicator of mosquito borne disease risk. However, larval surveys are known to correlate poorly with adult mosquito abundance, and have a variable association with human infection risk [2–6].

Similarly, climate variables are often explored in models of *Aedes*-borne disease risk as predictors of adult mosquito abundance, and infection risk. While climate is known to influence mosquito development and abundance, the extent to which climate factors are associated with standard vector surveillance indices has not been well-quantified [7,8]. Hence, the value of routinely collected vector surveillance data versus climate data, or other epidemiological variables for use in predictive models of *Aedes*-borne disease requires further empirical evaluation. This is of particular importance given the growing public health importance of *Aedes*-borne diseases [9–11] and concern regarding expanded mosquito and disease distributions in response to climate change [12,13].

The South East Asian region bears a particularly high burden of *Aedes*-borne diseases, with increasingly frequent epidemics of dengue as well as chikungunya and others [14–17]. In this region, there is also limited data for the linkages between vector dynamics and infection risk. As a first step toward improving linkages between these data, we examined available vector surveillance and climate data across 65 city/municipality areas of the island of Mindanao between 2013 and 2018 –in a region of the Southern Philippines where no published data on vector surveillance exist, but where the population is vulnerable to regular outbreaks of *Aedes*-borne disease [18]. In this remote region of the country, reliable arbovirus incidence data were not available. However, we analysed the spatial and temporal trends within and between two longitudinal datasets that were available in this understudied region: routinely collected public health vector surveillance data, and freely available climate data for the same areas. We sought to assess potential links between *Aedes* mosquito dynamics and climate factors in this region, to gain insight into the relationships between these two contributors to *Aedes*-borne diseases.

## Methods

### Study location

**Fig 1:**
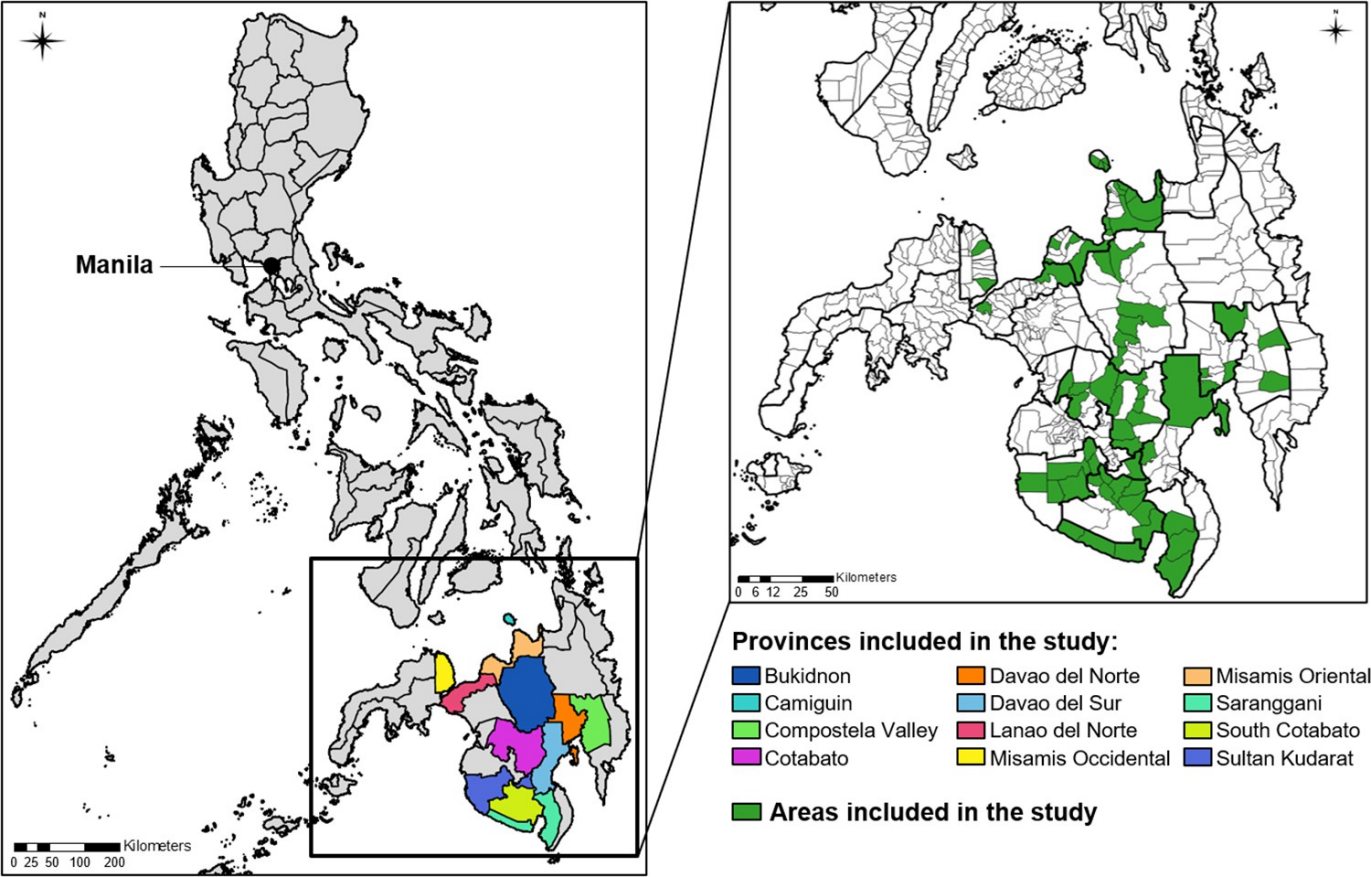
Map of the Philippines and the study regions. The Philippines is geographically divided into 87 provinces, with the capital city Manila located in the northern National Capital Region (NCR). Twelve southern provinces of the Mindanao geographic region where datasets were complete for the period 2013–2018 were included in our study, colour-coded on left. Inset, on right: the 65 areas within those provinces that were included in our study are indicated in green (comprising 14 cities and 51 municipalities). Provincial boundaries are marked by thick borders. Map data derived from the Humanitarian Data Exchange (https://data.humdata.org/)".

The Philippines is an archipelago of more than 7,500 islands, divided into 3 major geographic groups: the large northern island of Luzon, where the capital city Manila is situated, and the island groups of Visayas and Mindanao to the south (Fig 1). The climate is tropical, characterised by relatively high temperature (national mean annual temperature of 26.6°C), high humidity (monthly averages range between 71–85%) and abundant rainfall (mean annual total varies between 965 to 4,064mm) [19]. There are 2 main weather seasons: the dry season from December to May, and the rainy season from June to November. The Philippines is susceptible to climate-related disasters, such as tropical cyclones, and heavy precipitation and flooding from tropical storms, with the potential for increased communicable disease risks following these disasters [20–22].

Mindanao is the second largest and southernmost archipelago in the Philippines with a multi-ethnic population of 21.8 million (22% of the Philippine population) [23]. This region has a consistently hot and wet climate year-round, and is vulnerable to climate-induced disasters which pose risks to both health and the economy [24,25]. Socioeconomic levels are generally lower in Mindanao than in the rest of the country and the dominant economic activities are agriculture and fisheries [26]. While previously covered in dense tropical forest, much of the landscape has been converted to plantation agriculture, mainly pineapples and bananas [27,28]. The region reports regular outbreaks of dengue, often occurring in two seasonal peaks per year [18]. Zika virus infections have also been reported [16].

### Mapping files

Shapefiles delineating geographical boundaries for the Philippines were collected at administrative levels 2 (province) and 3 (city/municipality) from the Humanitarian Data Exchange, developed by the UN Office for the Coordination of Humanitarian Affairs [29]. All maps were created using ArcGIS Geographical Information System software (version 10·5, Esri USA).

### Entomological surveillance data

Larval surveillance for *Aedes* species was conducted through a joint effort by the Philippines Department of Health (DoH) and the Research Institute for Tropical Medicine (RITM), Manilla. The RITM serves as a National Reference Laboratory for *Aedes*-borne diseases, and monitors implementation of response strategies. Household entomological surveys were conducted as part of routine surveillance to investigate the occurrence of notified dengue epidemics, and to support the deployment of vector control activities. Surveys took place in 563 villages of the southern Philippines during 49/69 months between February 2013 –October 2018. Villages surveyed were chosen from selected cities and municipalities where dengue outbreaks were notified. The timing and location of surveys were dependent on when/where dengue outbreaks or clusters were identified by the DoH, and the human resources available at the time to conduct surveys. Hence, the number of times that a particular village was surveyed was variable, and there were no ‘control’ surveys conducted in locations without identified dengue outbreaks. The DoH consider an outbreak as an increase in registered cases in a given location for 2 consecutive weeks, while a cluster of cases occurs where a given location reports at least 3 dengue cases in 4 consecutive weeks [30].

A minimum of 100 houses per village were searched on each survey date, selected through random sampling of houses in each village. Random households were selected by dividing the total number of households present in the village by 100 to calculate the interval at which houses would be sampled, e.g. where the interval was seven, every seventh house would be visited for sampling. Experienced collectors surveyed water-holding containers both indoors and outdoors of the selected houses for evidence of *Aedes* larval breeding using standard methods [1], collecting all larvae and pupae found. The number of people resident in each house was recorded by asking a householder to report the number of people who had slept in the house the night prior to the survey. Larvae and pupae collected from each house, or each container, were counted and confirmed as either *Aedes aegypti* or *Ae*. *albopictus* by experienced local entomologists by rearing larvae to 4^th^ instar and rearing pupae to adults, and using illustrated identification keys. Larval indices for these two species (combined) were then calculated, including:

$$House\;Index\;(HI)=\frac{number\;of\;houses\;with\;larvae\;or\;pupae}{total\;houses\;surveyed}\;x\;100$$

$$Container\;Index\;(CI)=\frac{number\;of\;containers\;with\;larvae\;or\;pupae}{total\;containers\;surveyed}\;x\;100$$

$$Breteau\;Index\;(BI)=\frac{number\;of\;containers\;with\;larvae\;or\;pupae}{total\;houses\;surveyed}\;x\;100$$

$$Pupal\;productivity\;Index\;(PPI)=\frac{number\;of\;pupae}{number\;of\;persons\;resident\;in\;houses\;surveyed}\;x\;100$$

The DoH vector-borne disease control program in the Philippines uses HI≥ 5%, BI≥ 20 and PPI≥ 1% as thresholds for when vector control activities are required [30]. These include risk communication, social mobilisation and insecticide use–insecticide misting and/or spraying is generally only conducted in locations identified as a dengue cluster. Data on specific vector control activities conducted in response to entomological survey data collected were not available as part of this study.

### Climate data

Monthly climate data for precipitation, maximum & minimum temperature were extracted from the TerraClimate dataset [31] for the entire Philippines at administrative level 2 (city level), for the period January 2013 –December 2018. For each of the 65 areas where household larval surveillance occurred, these climate data were matched to the specific 49 months where larval surveillance was undertaken, and to the previous one and two months prior to each monthly survey, to allow for a 1- and 2-month lag period in analysing the association of climate variables with the presence of larvae.

### Data analysis

Household survey data were assessed for completeness and only locations with complete data for all survey variables were included. The number of villages, municipalities and cities surveyed in each province in any given month was variable, as these were selected based on availability of local staff and resources. Because of this variability, we aggregated the survey data for each village to city/municipality level and averaged each of the vector indices per location for each survey month. We then plotted temporal (monthly) trends in each of the vector indices by city/municipality (n = 65), province (n = 12), and across the whole study region using Excel. We then summarised spatial patterns (mean values) in each index by city/municipality.

Temporal and spatial trends in mean monthly values for each climate variable (maximum temperature, minimum temperature, precipitation) were summarised by province and city/municipality, respectively. Relationships between monthly entomological variables were then assessed for all 65 areas by conducting linear regression analyses between vector indices and climate variables. For this, monthly values for vector indices from each area were log transformed and matched with climate variables from the same city in the same month, and with climate variables measured one and two months prior to the household surveys. Correlations between the four different indices and climate variables for the three time points were determined using Spearman correlations. All analyses were conducted using Statistical Package for the Social Sciences (SPSS) Statistics software (IBM New York USA; version 23).

## Results

### Temporal and spatial trends in vector density indices

**Fig 2:**
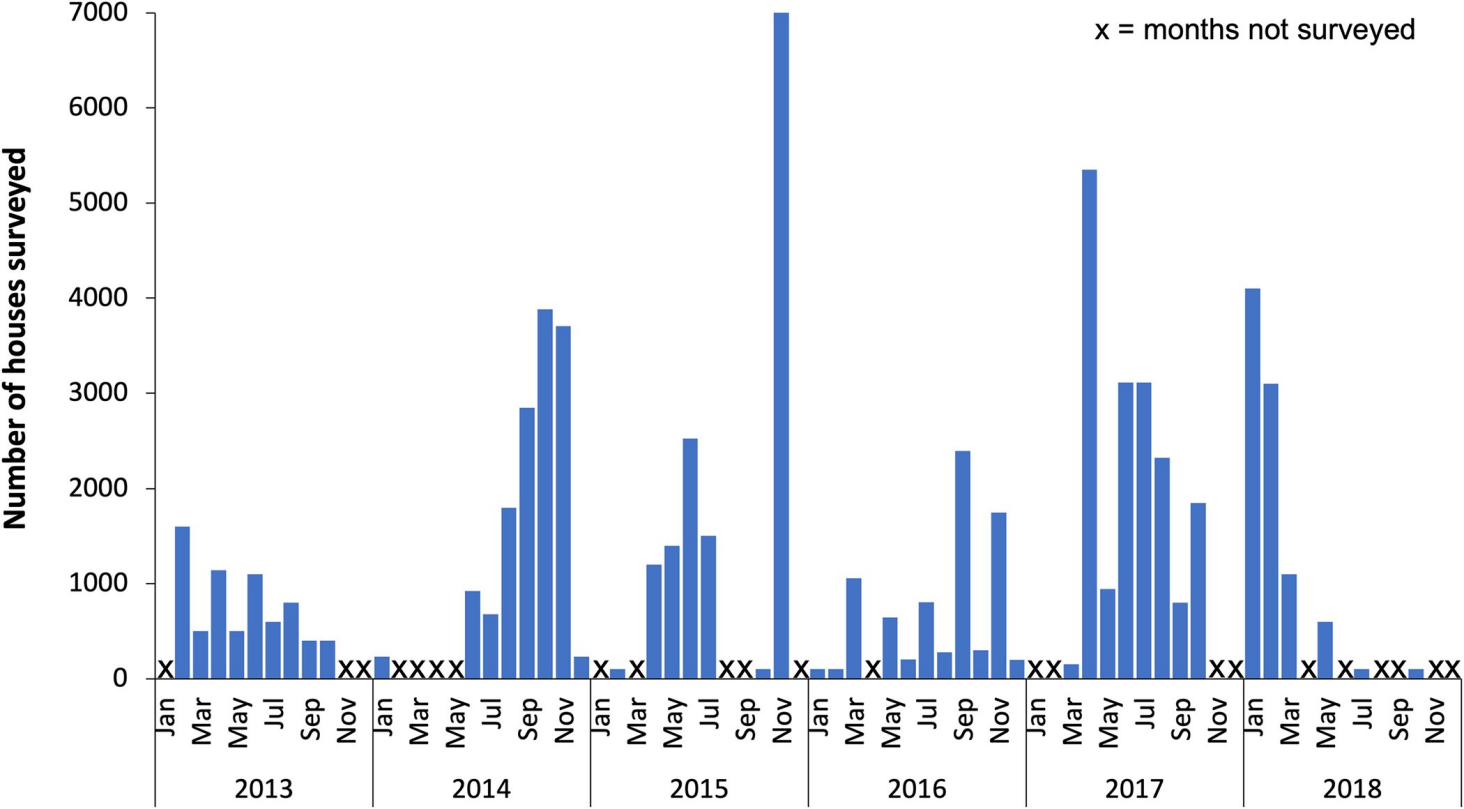
Temporal pattern of house surveys across the southern Philippines, 2013–2018. The monthly trend in number of houses surveyed for mosquito larvae across the southern Philippines during the 6-year period. Surveys varied in number and location each month depending on the number and duration of dengue outbreaks reported, with a total of 714 surveys conducted across the 563 villages of the 65 cities/municipalities in 49/69 months between Feb 2013 and Oct 2018.

A total of 714 household surveys were conducted during 2013–2018. This included 277,279 containers surveyed within 69,878 houses in 563 villages, within 65 cities/municipalities (14 cities and 51 smaller municipal areas) and 12 provinces (Table 1). See S1 Table for a complete list of locations. Overall, 10% of houses and 4% of all containers surveyed contained mosquito larvae or pupae (Table 1). The number of houses surveyed each month during the study period is shown in Fig 2.

**Table 1:** Summary of inspections conducted and their findings during the 6-year period: 2013–2018.

Provinces	Number of Municipalities (& cities) surveyed	Number of villages surveyed	Total no. houses surveyed	Total houses with larvae/ pupae (%)	Total no. containers surveyed	Total no. containers with larvae/ pupae (%)	Total no. people resident*	Total no. pupae identified
Bukidnon	5 (1)	70	11,634	1,354 (12%)	55,448	3,804 (7%)	53,541	28,879
Camiguin	4 (0)	58	8,262	129 (2%)	8,702	277 (3%)	25,952	21
Compostela Valley	3 (0)	6	2,800	222 (8%)	14,800	292 (2%)	9,480	173
Cotabato	9 (1)	41	4,126	930 (23%)	26,589	1,444 (5%)	19,887	965
Davao del Norte	0 (3)	46	7,001	643 (9%)	17,488	897 (5%)	23,984	343
Davao del Sur	1 (1)	128	10,354	858 (8%)	25,023	1,105 (4%)	37,162	102
Lanao del Norte	1 (1)	8	871	175 (20%)	663	215 (32%)	4,037	31
Misamis Occidental	0 (2)	25	4,700	479 (10%)	17,345	684 (4%)	19,785	543
Misamis Oriental	10 (2)	83	11,028	970 (9%)	45,199	1,181 (3%)	32,342	1,606
Saranggani	6 (0)	16	1,603	360 (22%)	26,452	589 (2%)	7,232	878
South Cotabato	6 (2)	65	5,799	773 (13%)	25,339	1,222 (5%)	25,697	598
Sultan Kudarat	6 (1)	17	1,700	367 (22%)	14,231	659 (5%)	7,929	429
**Total**	**65**	**563**	**69,878**	**7,260 (10%)**	**277,279**	**12,369 (4%)**	**267,028**	**34,568**

* Equal to the total number of people who slept in the houses surveyed the previous night

**Fig 3:**
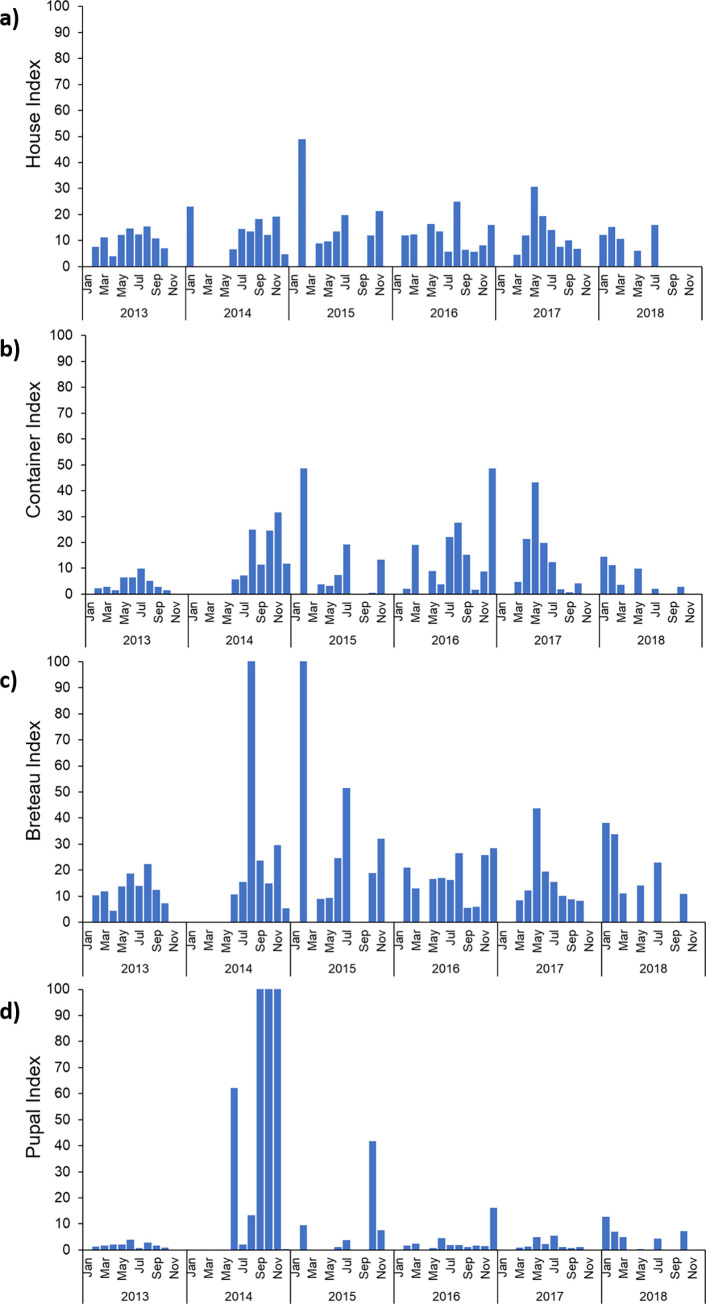
Temporal trend in vector indices, 2013–2018. Monthly averages per city are shown for each vector index across the 49 months surveyed: **a)** House Index, **b)** Container Index, **c)** Breteau Index and **d)** Pupal Productivity Index.

**Fig 4:**
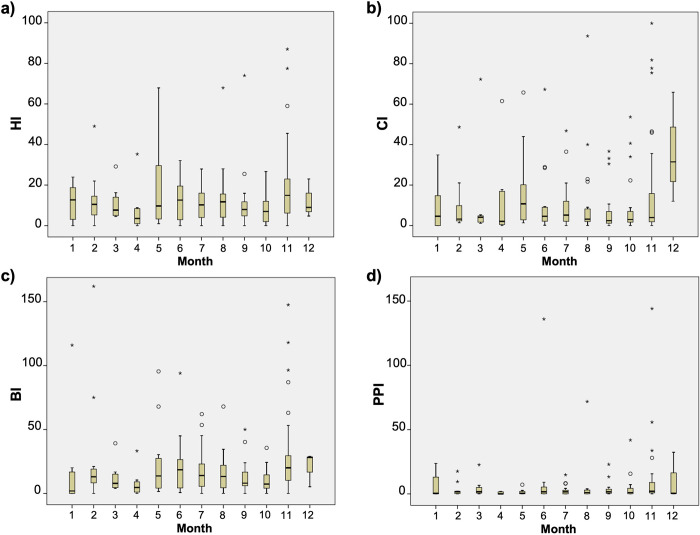
Monthly trend in vector indices across city/ municipality areas, 2013–2018. Monthly averages of **a)** House Index, **b)** Container Index, **c)** Breteau Index and **d)** Pupal Productivity Index are shown for all areas where data was collected between 2013 and 2018. Month numbers 1–12 on the x axis correspond to the months January-December.

Monthly indices varied substantially between cities/municipalities, with no apparent seasonal pattern present in HI, BI, CI or PPI (Fig 3). The average monthly house index per city/municipality ranged from 5 to 50 (mean = 15), the container index between 2 and 49 (mean = 16), and Breteau index between 4 and 162 (mean = 24). Pupal Productivity Index values were below 5 for most survey months, however there were distinct spikes in the average PPI in mid-late 2014 and 2015 (mean = 49 overall) (Figs 3 and 4). During these months, average PPI values increased to between 6 and 490 within 18 cities/municipalities across 5 provinces (mean = 388). The highest of these PPI values were recorded in 10 cities within 2 neighbouring northern provinces: Misamis Oriental and Bukidnon. In mid-2014, there was also a notable spike in BI, along with a second peak in early 2015, when relative increases occurred across all 4 indices (Fig 3).

**Fig 5:**
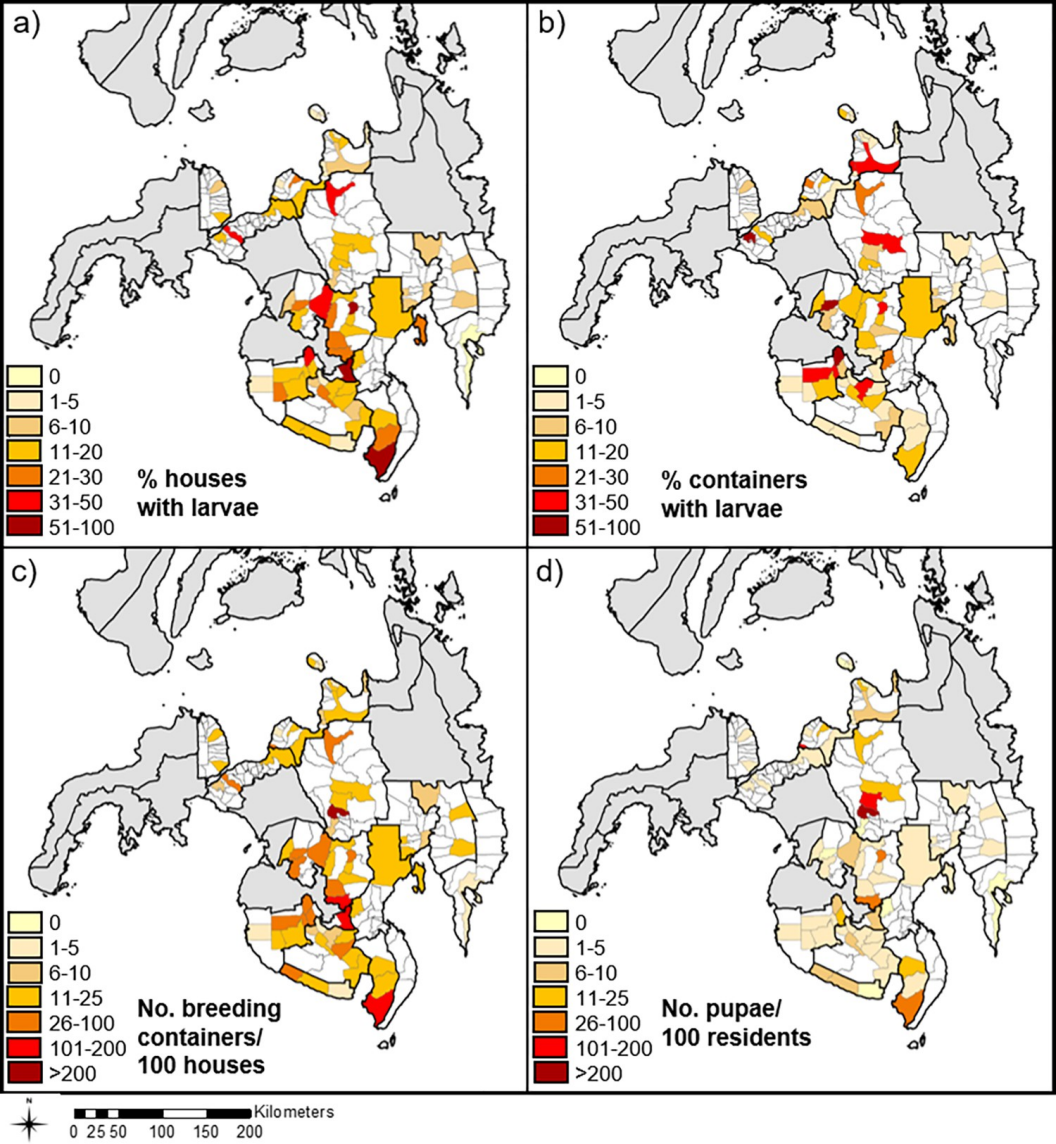
Spatial pattern of vector surveillance indices across the southern Philippines, 2013–2018. Mean monthly values for four vector surveillance indices are shown across 65 areas of the southern Philippines for the 6-year period. **a)** House Index, **b)** Container Index, **c)** Breteau Index and **d)** Pupal Productivity Index. The relative breeding intensity are shown by graduated colours. Areas not surveyed are indicated in white. Map data derived from the Humanitarian Data Exchange (https://data.humdata.org/)".

The spatial patterns for vector indices were also variable across areas, with similar trends in HI, BI and CI, and a more distinct pattern observed for the PPI (Fig 5). Across all survey locations, mean HI, BI and PPI values were above the designated thresholds for vector control in 76%, 40%, and 62% of survey months, respectively (S1 Table). The number of surveys conducted in any one location were variable, the data collected from each village sometimes also varied (e.g., data for one of the indexes was omitted) and hence rates of above-threshold values for indices also varied with location. For example, the number of surveys conducted in any one location was anywhere between 1 and 15 surveys (S1 Table), and for 28 of the 65 sampled locations (43%) only a single survey took place.

In locations where multiple surveys were conducted (≥3 surveys, n = 25/65 locations), the mean proportion of months where index values were above vector control thresholds were 67% for HI, 30% for BI and 54% for PPI (S1 Table). A small number of locations recorded above-threshold values in each index simultaneously across multiple (≥3) survey months. These included 7 locations across 4 provinces: 3 in Bukidnon province (the City of Valencia, the municipality of Manolo Fortich and the municipality of Maramag), 2 in South Cotabato province (General Santos City and Tampakan municipality); 1 in Misamis Oriental province (City of El Salvador); and 1 in Misamis Occidental (Ozamiz City) (S1 Table).

### Associations between climate variables and vector indices

During the study period, the 65 study areas recorded an average monthly minimum temperature of 21°C (compared to 23°C nationally), average maximum temperature of 31°C (compared to 31°C nationally) and a mean monthly precipitation level of 176mm (compared to 213mm nationally). The mean temporal trend for each climate variable across the study areas is shown in S1 Fig The monthly minimum temperature was consistent across all areas throughout each year, with seasonal fluctuations more evident for maximum temperature and precipitation. Maximum temperature peaked consistently between March and May each year, while precipitation levels fluctuated throughout the year, with high rainfall often occurring in January, June and September.

**Fig 6:**
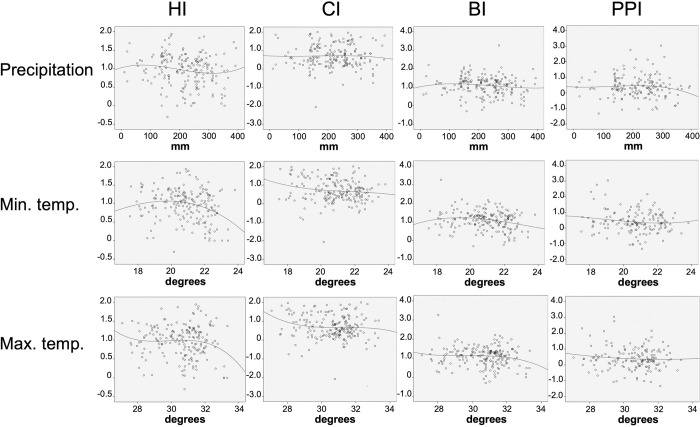
Relationship between vector density indices and climate variables, 2013–2018. Monthly values for each *Aedes* vector index: House Index (HI), Container Index (CI), Breteau Index (BI) and Pupal Productivity Index (PPI) were correlated with monthly precipitation, monthly minimum temperature (min. temp.) and monthly maximum temperature (max. temp.) across the 65 areas.

Using linear regression modelling, monthly averages for each vector index and each climate variable were compared for each city/municipality. Low magnitude, non-linear correlations were observed between the vector surveillance indices and climate variables (Fig 6). Minimum temperature showed small yet significant associations with the vector indexes HI, CI and BI when sampling months were matched to the same climate month (HI: R^2^ = 0.038, β = 2.269, p = 0.007; CI: R^2^ = 0.029, β = 2.292, p = 0.018; BI: R^2^ = 0.034, β = 2.551, p = 0.011), and when matched to the previous 1-month and 2-months (1-month lag values were HI: R^2^ = 0.033, β = 2.154, p = 0.013; CI: R^2^ = 0.021, β = 2.076, p = 0.045; BI: R^2^ = 0.032, β = 2.470, p = 0.014; and 2-month lag values were HI: R^2^ = 0.026, β = 1.986, p = 0.028; CI: R^2^ = 0.013, β = 1.729, p = 0.128; BI: R^2^ = 0.019, β = 2.124, p = 0.060). Maximum temperature showed a weak correlation with PPI with a 2-month lag (R^2^ = 0.031, β = 3.082, p = 0.032) but not with any other vector index. The PPI showed no other significant association with other climate variables tested. Rainfall was weakly correlated with HI when matched to the same sampling month (R^2^ = 0.023, β = 1.145, p = 0.04), but was not significantly correlated with any other index when comparing 1- or 2-month lagged precipitation data.

## Discussion

To improve preventive strategies for *Aedes*-borne diseases, increased understanding of factors influencing vector abundance is essential, especially in light of potential changes in mosquito-borne disease risks associated with climate change. We explored trends in routine *Aedes* vector surveillance indices in 65 areas of the southern Philippines, and compared these data with climate data from the same areas to assess the relationships between the two. We found that spatial and temporal patterns between entomological surveillance indices were similar, with no apparent seasonal trend. However, the PPI had a unique spatial and temporal pattern compared to the other indices. A small proportion of locations recorded above-threshold values across all entomological indices in multiple surveys. Each vector index showed variable, weak correlations with different climate variables when matched to the same survey months, or to the 1 or 2 months prior to the surveys.

Our findings were likely influenced by several complex factors which drive larval development and which operate at different geographic levels. For example, variation in the availability of breeding containers, microclimate suitability, land use and vegetation types present and residential water-use practices, which may have differential effects on *Aedes* breeding [32–34]. In addition, the larval surveillance techniques used may not capture the full range of breeding habitats for all species, especially for *Ae*. *albopictus* [35,36]. The ability to sample all visible indoor and outdoor water-holding containers also depends on the level of access provided by residents, and may limit access to more cryptic breeding sites. Hence, while the rising and falling patterns observed in the larval and pupal indices across the study period could reflect the timing of changes in broad scale climate variables, the trends observed were likely also influenced by human behavioural and socioeconomic factors such as the presence of artificial breeding containers around the home and/or variation in access to breeding sites during surveys [37–40].

Similarly, human behaviour, sampling biases and differences in geographic scale of data types may also explain the relatively weak correlations we observed between city-level climate variables and household-level entomological indices. Although we were unable to estimate the relative strengths of these different influences on our results, previous studies suggest that the impact of human behaviour alone can have significant impacts on trends in larval and pupal indices, through providing breeding containers such as discarded tyres and plastic containers around homes [40–42]. Variation in the land use types and vegetation levels surrounding the residential areas surveyed is also likely to have influenced trends in indices between locations [34]. Previous studies have found that surrounding vegetation types and urbanisation levels to have substantial effects on adult *Aedes* species abundance, through providing a resting place and sugar source for adults, although the effect can be highly dependent on broader characteristics of the sampling location as well such as human socioeconomic and behavioural factors [43,44]. Each of these influences would be a useful variable to explore in future studies–particularly in the seven locations that consistently recorded above-threshold values in indices across multiple surveys. It was notable that these locations were geographically widespread, and included both larger cities and smaller municipalities, and may have differing socioeconomic and environmental influences on both microclimate and larval development.

Despite the known strong influence of climate on mosquito development [45–47], we found that broad-scale climate variables had only a small effect on the presence of immature *Aedes* mosquitoes at the household level. This may be a result of the type and scale of the climatic and entomological data used. The climate dataset used in this study was limited in both the variables included and their spatial and temporal detail, and this in turn limits the conclusions we can draw from our analyses. In a tropical environment such as the southern Philippines, where the variation in daily temperatures is low, it may be important to capture finer-scale variations in climate variables to properly assess their influence on different stages of mosquito development. It is also possible that relationships between climate and vector-borne disease spread may have more to do with weather impacts on adult mosquito populations than on larval populations, as studied here. Previous studies examining links between broad scale climate data and residential mosquito breeding have reported mixed findings, and are further impacted by the influence of uncontrolled ecological and socio-economic factors on the presence of mosquito larvae and their development into adults [48,49]. Broad scale climate data does not capture microclimate in urban habitats, which is increasingly recognised as crucial and yet is rather disconnected to weather station data [32,33,50]. However, our study provides useful baseline analyses that can be further built upon in future studies; for example, through the measurement of climate variables at finer spatial and temporal scales.

Evidence for the value of entomological indices as predictors of *Aedes*-borne disease outbreaks are inconclusive [3,51,52]. For example, a Vietnamese study found a significant correlation between all entomological and climate variables tested and dengue incidence [53], while an Indonesian study showed traditional vector indices were of limited value as indicators of dengue risk [54]; instead, adult species composition was more indicative of dengue endemicity. An alternative approach was proposed in a Colombian study which found that a landscape ecological niche model was more useful to predict dengue case rates than the BI, which had no relationship to case rates [55]. However, few modelling studies have compared vector indices with climate variables, and most examine this indirectly by comparing climate factors and mosquito-borne disease incidence without considering vector dynamics specifically. Hence, there is insufficient empirical or theoretical data on the best use of both climate and entomological indices as predictors of human disease risk.

In terms of disease prevention, it was notable that the mean monthly values for larval indices were regularly above the nationally recommended thresholds for triggering vector control responses by the Department of Health. In particular, HI was above 5% in the majority (76%) of survey months, and a number of geographically widespread locations consistently recorded above-threshold values. This implies that substantial resources and human resource capacity would be required for vector control programs to implement vector control responses based on current thresholds of entomological indices. Dengue control guidelines in the Philippines state that in response to either an increase in dengue cases, or in the case of larval indices being above the designated thresholds, response measures undertaken may include: indoor and outdoor insecticide spraying (residual spraying and misting), community clean-up campaigns (larviciding and larval source reduction), and follow up larval surveys. Larval source reduction is often the mainstay of control strategies; however, it is not simple to implement given the range of habitats and breeding containers that can be utilised by *Aedes* mosquitoes. Insecticide misting or spraying is generally only performed in dengue outbreak areas where there is an increase in cases registered for 2 consecutive weeks. Indoor residual insecticide spraying (within homes or schools) is a method more well-supported by evidence of impact [56,57], yet is conducted in the Philippines only when an outbreaks continues for 4 consecutive weeks.

The specific vector control interventions that took place based on the larval survey data presented here are not known, but it is likely that at a minimum dengue risk communications or community clean up campaigns of mosquito breeding sites took place in at least some of the study areas. These interventions may have had impacts on if/when areas surveyed in a particular month were re-surveyed (or not) in subsequent months. Therefore, it can be assumed that the trends observed in entomological indicators in our study likely do not reflect the impact of climate factors alone, and this might partly explain the small yet significant relationships we observed between the entomological indicators and climate variables. Similarly, the impact that vector control activities may have had on dengue incidence in these villages is not known. In the absence of this additional data for vector control responses and dengue incidence, it is difficult to ascertain how useful larval and pupal indices are as indicators for vector control responses that aim to prevent *Aedes*-borne pathogens transmitted by adult mosquitoes. Future studies to ascertain the value of larval and pupal surveillance should include additional data on the timing and location of vector control responses, and would be further strengthened by the addition of adult mosquito surveillance and dengue incidence data obtained from the same locations.

Despite the lack of scientific consensus on the value of entomological indices to guide disease prevention efforts, the collection of larval surveillance data remains a routine public health measure in many countries endemic for *Aedes*-borne diseases. Guidance on collection of larval surveillance data was provided by the World Health Organization in its 2009 dengue control guidelines [1]; however, the advice has since shifted towards a focus on adult mosquitoes, which may be more accurately indicated by the presence of pupae rather than larvae [58]. Evidence suggests that targeting breeding sites producing pupae is a more efficient strategy than attempting to treat all breeding sites [59], so this could be a useful consideration in re-orienting surveillance activities of control programs.

Practical and resource limitations might also suggest raising the threshold for public health responses based on PPI values, for example from 1% to 5%. In our dataset, the number of survey months where any location had a PPI >1% was 62% (versus 25% using a 5% threshold), and the number of locations that were above the 1% threshold at any one time was 49/65 (versus 27/65 using 5%). However, evidence for the impact of a change such as this on *Aedes*-borne disease rates would need to be carefully evaluated before implementation. The value of using surveillance data of adult vector species as a vector control indicator would also be useful to investigate [60]. Further, the necessary reductions in adult or immature mosquitoes required to significantly reduce disease incidence rates is unknown, despite vector control being the dominant prevention strategy for *Aedes*-borne diseases. These are important areas for future research which were outside the scope of this study.

Our study was limited by working with operational data sets which were generated for public health purposes rather than for research, and are variable and opportunistic by nature. These limitations include variation in survey locations over time, and gaps in survey months, which hindered a fuller assessment of seasonal trends and comparisons of means. We also did not have access to data for abundance of individual *Aedes* species, so could not ascertain whether our findings reflected populations of *Ae*. *aegypti*, *Ae*. *albopictus* or both species. The presence of additional species of importance was also not considered here. Species-specific data is critical to collect for future studies, alongside epidemiological data on *Aedes*-borne disease rates, as it could further assist the targeting of vector control to specific vector habitat types.

We suggest that the value of specific vector surveillance indices and climate variables in guiding disease prevention and control efforts should be further explored. Future research would ideally incorporate both immature and adult mosquito abundance for each *Aedes* species, data on *Aedes*-borne disease incidence and vector control practices, along with finer-scale climate, or microclimate, variables. Additional influencing factors to explore might include socio-environmental factors (such as urbanisation level, household water storage and larval control practices, and local habitat suitability for *Aedes*). A more comprehensive analysis of these data may assist further evaluation of the value of current vector surveillance indices in *Aedes* surveillance and control efforts.

## Conclusions

This study presents longitudinal real-world surveillance data on *Aedes* mosquitoes in a dengue outbreak-prone area. The data provide a snapshot of trends in larval and pupal indices in the context of ongoing outbreaks, and their relationships with climate variables. Overall, our findings demonstrate that household surveillance indices for HI, CI, BI and PPI are poorly correlated with city-level climate variables. Whether larval indices are reliable measures to direct vector control responses, or whether they have value as predictors of *Aedes*-borne disease risk requires further research. Future studies should ideally use a combination of entomological and epidemiological data, and include broader consideration of influencing factors such as socio-environmental factors. In particular, future research should aim to identify the most effective *Aedes* surveillance methods to inform vector control measures, and to quantify the impact of *Aedes* vector surveillance and control on reducing the burden of *Aedes*-borne diseases.

## Supporting information

S1 Table List of study locations and occurrence of above-threshold values for entomological indices. (DOCX)

S1 Fig Temporal trend in climate variables across cities, 2013–2018. Average monthly values for each of **a)** minimum temperature, **b)** maximum temperature and **c)** precipitation are shown for the 65 areas included in the study for the 7-year study period.
(TIF)
