# Supplementary material for: Climate variability and Aedes vector indices in the southern Philippines: An empirical analysis
Source: PLoS Negl Trop Dis. 2022 Jun 14;16(6):e0010478. doi: 10.1371/journal.pntd.0010478 (PMC9197058; doi:10.1371/journal.pntd.0010478)
Supplement: S1 Table — (DOCX) [file pntd.0010478.s001.docx]

**S1 Table. List of study locations and occurrence of above-threshold values for entomological indices**

| **Province** | **City*/municipality** | **Population  (2015 census)** | **No. months HI>5%**  **(total months surveyed)** | **Proportion of months HI> 5%** | **No. months BI>20**  **(total months surveyed)** | **Proportion of months BI >20** | **No. months PPI>1%**  **(total months surveyed)** | **Proportion of months PPI> 1%** | |
| --- | --- | --- | --- | --- | --- | --- | --- | --- | --- |
| Bukidnon | City of Valencia*^ | 192,993 | 10 (12) | 83% | 4 (12) | 33% | 11 (12) | 92% | |
| Bukidnon | Don Carlos | 66,959 | 3 (3) | 100% | 2 (3) | 67% | 3 (3) | 100% | |
| Bukidnon | Kadingilan | 33,778 | 1 (1) | 100% | 0 (1) | 0% | 0 (1) | 0% | |
| Bukidnon | Libona | 43,969 | 1 (1) | 100% | 1 (1) | 100% | 1 (1) | 100% | |
| Bukidnon | Manolo Fortich^ | 100,210 | 7 (7) | 100% | 6 (7) | 86% | 5 (7) | 71% | |
| Bukidnon | Maramag^ | 102,089 | 4 (9) | 44% | 3 (9) | 33% | 4 (9) | 44% | |
| Camiguin | Catarman | 16,798 | 3 (7) | 43% | 2 (7) | 29% | 0 (7) | 0% | |
| Camiguin | Guinsiliban | 6,281 | 0 (5) | 0% | 0 (5) | 0% | 0 (5) | 0% | |
| Camiguin | Mahinog | 14,038 | 0 (6) | 0% | 0 (6) | 0% | 0 (6) | 0% | |
| Camiguin | Sagay | 12,626 | 0 (5) | 0% | 0 (5) | 0% | 0 (5) | 0% | |
| Comostela Valley | Compostela | 87,474 | 1 (1) | 100% | 0 (1) | 0% | 1 (1) | 100% | |
| Comostela Valley | Laak | 73,874 | 4 (8) | 50% | 1 (8) | 13% | 1 (3) | 33% | |
| Comostela Valley | Maragusan | 60,842 | 2 (3) | 67% | 0 (3) | 0% | 2 (3) | 67% | |
| Cotabato | Antipas | 25,304 | 1 (1) | 100% | 1 (1) | 100% | 1 (1) | 100% | |
| Cotabato | Carmen | 95,921 | 1 (1) | 100% | 1 (1) | 100% | 1 (1) | 100% | |
| Cotabato | City of Kidapawan* | 140,195 | 3 (3) | 100% | 1 (3) | 33% | 1 (3) | 33% | |
| Cotabato | Kabacan | 89,161 | 1 (1) | 100% | 1 (1) | 100% | 1 (1) | 100% | |
| Cotabato | Libungan | 48,768 | 1 (1) | 100% | 1 (1) | 100% | 0 (1) | 0% | |
| Cotabato | Midsayap | 151,684 | 2 (3) | 67% | 2 (3) | 67% | 2 (3) | 67% | |
| Cotabato | M'Lang | 95,070 | 2 (2) | 100% | 2 (2) | 100% | 2 (2) | 100% | |
| Cotabato | Pigkawayan | 66,796 | 1 (1) | 100% | 0 (1) | 0% | 1 (1) | 100% | |
| Cotabato | President Roxas | 47,575 | 1 (1) | 100% | 0 (1) | 0% | 0 (1) | 0% | |
| Cotabato | Tulunan | 56,513 | 1 (1) | 100% | 1 (1) | 100% | 1 (1) | 100% | |
| Davao del Norte | City of Panabo* | 184,599 | 2 (3) | 67% | 0 (3) | 0% | 2 (3) | 67% | |
| Davao del Norte | City of Tagum* | 259,444 | 1 (1) | 100% | 0 (1) | 0% | 0 (1) | 0% | |
| Davao del Norte | Island Garden City of Samal* | 104,123 | 2 (2) | 100% | 2 (2) | 100% | 2 (2) | 100% | |
| Davao del Sur | Davao City* | 1,632,991 | 8 (9) | 89% | 2 (9) | 22% | 1 (8) | 13% | |
| Davao del Sur | Magsaysay | 19,019 | 1 (1) | 100% | 1 (1) | 100% | 0 (1) | 0% | |
| Lanao del Norte | Iligan City* | 342,618 | 2 (2) | 100% | 2 (2) | 100% | 1 (2) | 50% | |
| Lanao del Norte | Lala | 67,727 | 1 (1) | 100% | 0 (1) | 0% | 0 (1) | 0% | |
| Misamis Occidental | Ozamiz City*^ | 141,828 | 13 (15) | 87% | 2 (15) | 13% | 13 (15) | 87% | |
| Msamis Oriental | Balingoan | 11,051 | 4 (4) | 100% | 2 (4) | 50% | 4 (4) | 100% | |
| Msamis Oriental | Binuangan | 7,515 | 0 (3) | 0% | 0 (3) | 0% | 1 (3) | 33% | |
| Msamis Oriental | Cagayan de Oro City* | 675,950 | 5 (5) | 100% | 1 (5) | 20% | 3 (5) | 60% | |
| Msamis Oriental | City of El Salvador*^ | 50,204 | 6 (8) | 75% | 5 (8) | 63% | 8 (8) | 100% | |
| Msamis Oriental | Claveria | 48,906 | 3 (3) | 100% | 0 (3) | 0% | 3 (3) | 100% | |
| Msamis Oriental | Initao | 32,370 | 1 (3) | 33% | 0 (3) | 0% | 0 (3) | 0% | |
| Msamis Oriental | Jasaan | 54,478 | 0 (2) | 0% | 0 (2) | 0% | 2 (2) | 100% | |
| Msamis Oriental | Lugait | 19,758 | 1 (1) | 100% | 1 (1) | 100% | 1 (1) | 100% | |
| Msamis Oriental | Magsaysay | 53,876 | 0 (2) | 0% | 0 (2) | 0% | 2 (2) | 100% | |
| Msamis Oriental | Medina | 32,907 | 4 (4) | 100% | 2 (4) | 50% | 1 (4) | 25% | |
| Msamis Oriental | Oroquieta City* | 70,757 | 3 (4) | 75% | 2 (4) | 50% | 3 (4) | 75% | |
| Msamis Oriental | Talisayan | 24,505 | 0 (1) | 0% | 0 (1) | 0% | 0 (0) | 0% | |
| Msamis Oriental | Villanueva | 39,378 | 0 (1) | 0% | 0 (1) | 0% | 0 (1) | 0% | |
| Saranggani | Alabel | 80,359 | 1 (1) | 100% | 0 (1) | 0% | 1 (1) | 100% | |
| Saranggani | Glan | 118,263 | 1 (1) | 100% | 1 (1) | 100% | 1 (1) | 100% | |
| Saranggani | Kiamba | 61,058 | 2 (2) | 100% | 1 (2) | 50% | 2 (2) | 100% | |
| Saranggani | Maasim | 59,468 | 0 (1) | 0% | 0 (1) | 0% | 0 (1) | 0% | |
| Saranggani | Maitum | 44,595 | 2 (2) | 100% | 1 (2) | 50% | 1 (2) | 50% | |
| Saranggani | Malapatan | 76,914 | 1 (1) | 100% | 1 (1) | 100% | 1 (1) | 100% | |
| South Cotabato | Banga | 83,989 | 1 (1) | 100% | 1 (1) | 100% | 1 (1) | 100% | |
| South Cotabato | City of Koronadal* | 174,942 | 2 (2) | 100% | 0 (2) | 0% | 1 (1) | 100% | |
| South Cotabato | General Santos City*^ | 594,446 | 7 (8) | 88% | 4 (8) | 50% | 7 (8) | 88% | |
| South Cotabato | Norala | 46,642 | 1 (1) | 100% | 0 (1) | 0% | 1 (1) | 100% | |
| South Cotabato | Polomolok | 152,589 | 2 (2) | 100% | 0 (2) | 0% | 1 (2) | 50% | |
| South Cotabato | Tampakan^ | 39,525 | 4 (4) | 100% | 3 (4) | 75% | 3 (3) | 100% | |
| South Cotabato | Tantangan | 43,245 | 0 (1) | 0% | 0 (1) | 0% | 0 (1) | 0% | |
| South Cotabato | Tupi | 69,976 | 2 (2) | 100% | 1 (2) | 50% | 2 (2) | 100% | |
| Sultan Kudarat | Bagumbayan | 67,061 | 1 (1) | 100% | 0 (1) | 0% | 1 (1) | 100% | |
| Sultan Kudarat | City of Tacurong* | 98,316 | 2 (2) | 100% | 1 (2) | 50% | 2 (2) | 100% | |
| Sultan Kudarat | Columbio | 33,258 | 1 (1) | 100% | 1 (1) | 100% | 1 (1) | 100% | |
| Sultan Kudarat | Isulan | 90,682 | 2 (2) | 100% | 1 (2) | 50% | 1 (1) | 100% | |
| Sultan Kudarat | Kalamansig | 49,059 | 0 (1) | 0% | 0 (1) | 0% | 0 (1) | 0% | |
| Sultan Kudarat | Lambayong | 77,013 | 1 (1) | 100% | 1 (1) | 100% | 1 (1) | 100% | |
| Sultan Kudarat | Sen. Ninoy Aquino | 46,882 | 1 (1) | 100% | 0 (1) | 0% | 0 (1) | 0% | |
|  |  |  |  |  |  |  |  |  | |
| **Average proportion of months with above threshold values**  **for entomological indices** | | | | **76%** |  | **40%** |  | **62%** | |
| *Locations classified as cities rather than municipalities. City populations ranged between 50,204-1,632,991 (mean 333,100), while municipality populations were between 6,281-152,589 (mean 57,800).  ^Locations where above-threshold values were recorded in each index simultaneously across multiple (≥3) survey months. | | | | | | | | |  |
