# Supplementary figures and images for: Climate variability and Aedes vector indices in the southern Philippines: An empirical analysis
Source: PLoS Negl Trop Dis. 2022 Jun 14;16(6):e0010478. doi: 10.1371/journal.pntd.0010478 (PMC9197058; doi:10.1371/journal.pntd.0010478)

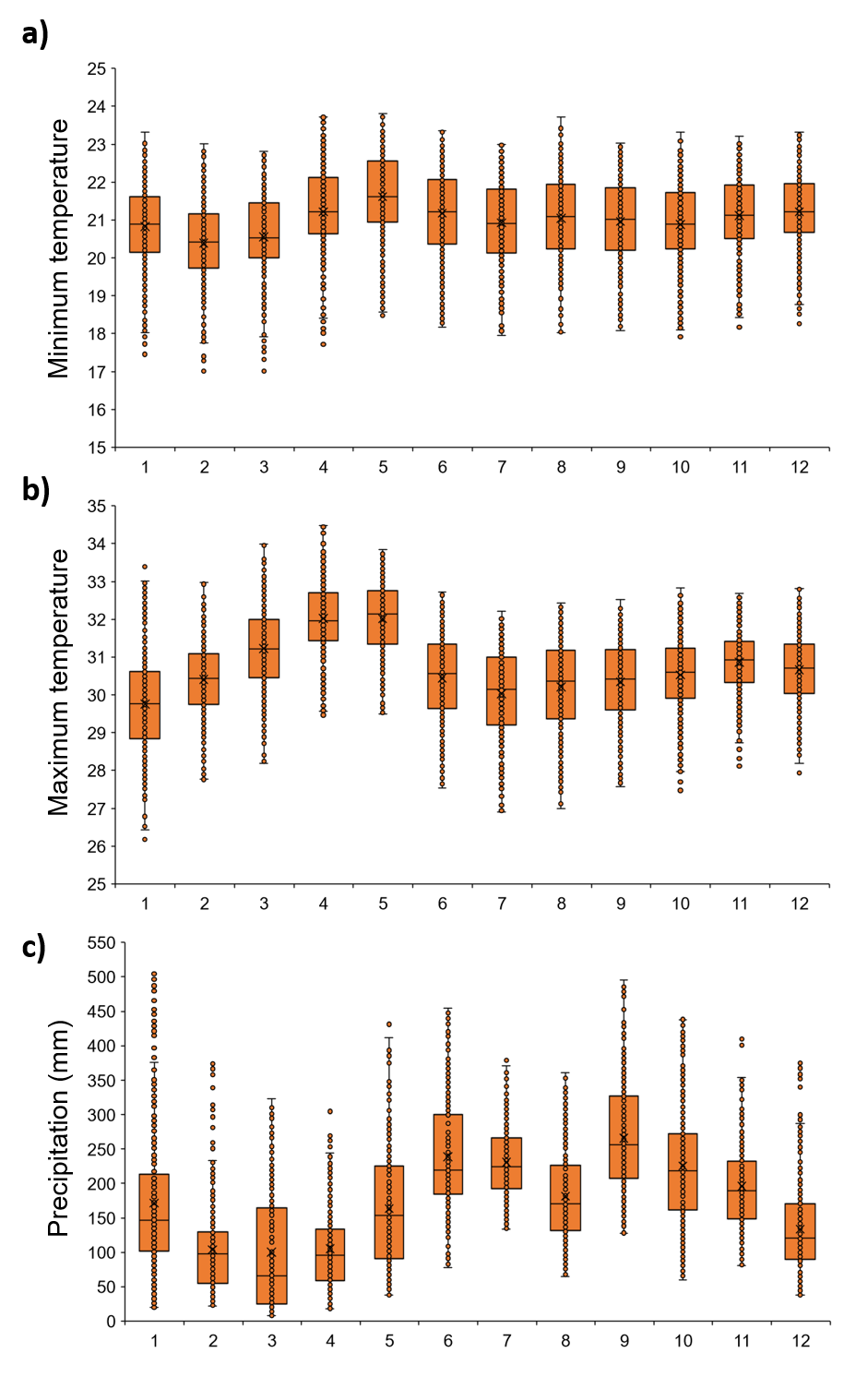

Supplement: S1 Fig — Average monthly values for each of a) minimum temperature, b) maximum temperature and c) precipitation are shown for the 65 areas included in the study for the 7-year study period. (TIF) [file pntd.0010478.s002.tif]
